# Radiobiotherapy in Osteosarcoma: A State-Based Educational Framework for Strategy Selection and Trial Design

**DOI:** 10.3390/curroncol33060342

**Published:** 2026-06-08

**Authors:** Srinivasan Vijayakumar, Shirley Lewis, Marc Matrana, Robert J. Vasquez, Anshul Singh, Nicholas Duesbery, Anderson B. Collier, Zoe Larned, Jennifer Barr, Wayne R. Orr, Mary R. Nittala, Vani Vijayakumar

**Affiliations:** 1 Department of Radiology, Ochsner Clinic Foundation and Xavier Ochsner College of Medicine, New Orleans, LA 70112, USA; 2 Department of Radiation Oncology, Kasturba Medical College, Manipal Academy of Higher Education, Manipal, India; shirley.salins@manipal.edu (S.L.); dr.anshulsingh@gmail.com (A.S.); 3 Cancer Care Advisors and Consultants, LLC, Ridgeland, MS 39157, USA; 4 Mississippi Comprehensive Cancer Control Program, Jackson, MS 39216, USA; 5 The Gayle and Tom Benson Cancer Center, Jefferson, LA 70121, USA; mamatrana@ochsner.org; 6 Precision Medicine, Ochsner Health, Jefferson, LA 70121, USA; 7 Physician Research Steering Committee and Physician Research Council, Lincoln, NE 68516, USA; 8 Clinical Cancer Research, Ochsner MD Anderson Cancer Center, New Orleans, LA 70121, USA; zlarned@ochsner.org; 9 Experimental Therapeutics, Ochsner Health, Houston, TX 77054, USA; 10 Hematology and Oncology, Xavier Ochsner College of Medicine (XOCOM), New Orleans, LA 70112, USA; 11 Faculty of Medicine, The University of Queensland Medical School, Brisbane, QLD 4006, Australia; 12 Pediatric Stem Cell Transplant and Adolescent and Young Adult Cancer and Survivor Programs, Ochsner Health, New Orleans, LA 70112, USA; rovasquez@ochsner.org; 13 Division of Pediatric Hematology-Oncology, Ochsner Children’s Hospital, New Orleans, LA 70118, USA; 14 Research, Ochsner Health, Jefferson, LA 70121, USA; 15 Ochsner Center for Outcomes Research, New Orleans, LA 70121, USA; 16 Division of Hematology/Oncology, Nemours Children’s Health, Jacksonville, FL 32207, USA; anderson.collier@nemours.org; 17 Hematology/Oncology, Ochsner Health, New Orleans, LA 70121, USA; 18 Department of Orthopaedic Surgery and Rehabilitation, Orthopaedic Oncology, University of Mississippi Medical Center, Jackson, MS 39216, USA; jbarr@umc.edu; 19 Department of Surgery, Surgical Oncology, University of Mississippi Medical Center, Jackson, MS 39216, USA; worr@umc.edu; 20 Department of Radiation Oncology, University of Mississippi Medical Center, Jackson, MS 39216, USA; 21 Nuclear Medicine, Diagnostic Radiology, Ochsner Health, New Orleans, LA 70115, USA; vani.vijayakumar@ochsner.org; 22 Ochsner Medical Center—New Orleans, New Orleans, LA 70121, USA

**Keywords:** osteosarcoma, radiobiotherapy, state-based decision-making, educational framework, hypothesis generation, stereotactic body radiotherapy, spatially fractionated radiotherapy, clinical trial design

## Abstract

**Background**: Osteosarcoma remains a biologically complex and clinically challenging malignancy, with survival gains plateauing despite decades of multimodal therapy incorporating surgery and cytotoxic chemotherapy. Unlike cancers in which mutation-centric precision oncology has yielded transformative advances, osteosarcoma is characterized by profound structural variation, copy number alteration dominance, and dynamic clonal evolution, limiting the effectiveness of single-target approaches. These realities motivate alternative strategy-level frameworks that better align treatment selection with evolving disease behavior. **Methods**: This narrative educational review synthesizes contemporary evidence from osteosarcoma biology, radiobiology, and translational oncology to propose a state-based framework for integrating radiotherapy—particularly stereotactic body radiotherapy (SBRT/SABR) and spatially fractionated radiotherapy (SFRT)—into osteosarcoma management and clinical trial design. Rather than relying solely on static anatomic stage, this framework emphasizes clinically actionable, time-varying state variables, including disease burden patterns (localized, oligometastatic, polymetastatic), tempo of progression, prior systemic response, and feasibility of complete local control. **Results**: Within this context, radiotherapy is presented not only as a local control modality but also as a hypothesis-generating biologic intervention, capable of perturbing tumor vasculature, inflammatory signaling, innate DNA-sensing pathways, and immune/myeloid programs in a dose-, fractionation-, and spatial-distribution-dependent manner. The review critically examines both the potential opportunities (e.g., local eradication, immune modulation) and limitations (e.g., rarity of abscopal responses, risk of unintended systemic signaling) of radiobiotherapy combinations, emphasizing the need for cautious interpretation and prospective validation. **Conclusions**: Finally, the article outlines practical implications for state-stratified, biomarker-embedded clinical trials, highlighting endpoints beyond conventional response criteria, including circulating tumor DNA dynamics, immune and myeloid signatures, and long-term patterns of disease progression. Overall, this review frames radiobiotherapy as an educational and investigational paradigm intended to support rational hypothesis generation, multidisciplinary decision-making, and learning-oriented trial designs in osteosarcoma, rather than as definitive clinical guidance.

## Simple Summary

Osteosarcoma is a rare and aggressive bone cancer that is especially difficult to treat when it returns or spreads. Standard staging shows where the cancer is, but it may not fully reflect how fast the disease is changing or whether all known tumors can realistically be controlled. This paper presents an educational, hypothesis-generating framework for “state-based” decision-making that also considers tumor burden, pace of progression, prior treatment response, and whether complete local treatment is feasible. It discusses how modern radiation approaches might help control visible tumors and influence the broader tumor environment. Importantly, these ideas are not yet established practice and need further testing in prospective clinical trials. The paper also suggests future trial approaches that could help refine treatment planning and improve research in osteosarcoma.

## 1. Introduction: Why Re-Thinking Osteosarcoma Therapy Is Necessary

Osteosarcoma is an aggressive primary bone malignancy that disproportionately affects adolescents and young adults [1,2,3]. Despite multimodality treatment combining surgery and multi-agent chemotherapy, outcomes for high-risk disease remain poor, and population-level survival gains have plateaued over recent decades [1,2,3,4]. Early multimodality strategies established the importance of pathologic response and postoperative adaptation [5,6]. Large and cooperative group experiences further demonstrated that histologic response—often defined using a ≥90% necrosis threshold—is a strong prognostic marker [7,8,9]. However, attempts to intensify therapy for poor responders have not reliably improved event-free survival, underscoring the need for new strategy-level approaches [8].

Recent observational data have also challenged long-standing assumptions regarding the optimal sequencing of neoadjuvant chemotherapy versus surgery-first approaches, suggesting this question may warrant renewed prospective evaluation [9,10]. Collectively, these realities highlight the need for innovation beyond incremental modifications of existing systemic regimens [2,3,4,8,9,10].

This educational review evolved from our recently published case report describing a chemo-nonresponsive telangiectatic osteosarcoma with oligometastatic recurrence managed with multimodality therapy, including SABR [9].

**Figure 1:**
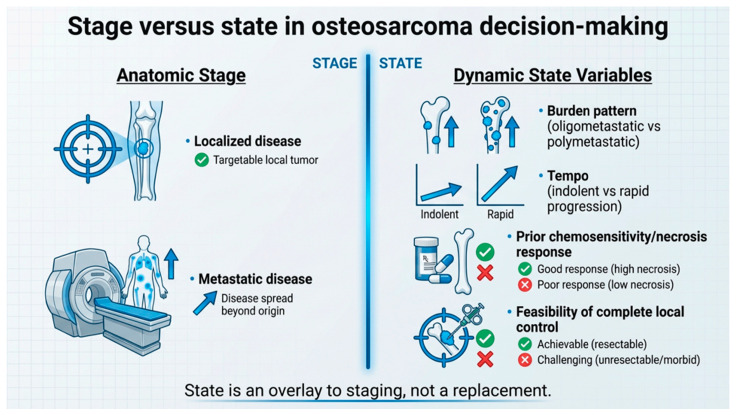
Stage-based versus state-based frameworks in osteosarcoma. This figure contrasts anatomic staging (e.g., localized vs metastatic disease) with a state-based framework that adds clinically actionable, time-varying features such as disease burden pattern (localized/oligometastatic/polymetastatic), tempo of progression, prior treatment response (including necrosis), and feasibility of complete local control. The goal is to support strategy selection and trial stratification rather than to replace standard staging systems.

To help distinguish conventional stage-based thinking from the dynamic, strategy-oriented approach used throughout this review, Figure 1 and Table 1 provide a concise visual and side-by-side comparison.

This review is intended as a narrative, educational, and hypothesis-generating synthesis of the current literature rather than as a practice guideline. Its purpose is to examine osteosarcoma through a state-based decision-making framework that integrates disease burden, tempo, prior treatment response, and feasibility of complete local control. Within that framework, the article explores how radiobiotherapy concepts may inform strategy selection and identifies areas in which prospective, biomarker-embedded clinical trials are needed. The sections that follow are therefore designed to highlight current knowledge gaps, organize the field conceptually, and suggest practical directions for future research.

## 2. Article Roadmap and Intended Audience

This review synthesizes osteosarcoma biology and radiobiology into a state-based framework for strategy selection and trial design. The figures and tables are intended as teaching schematics that translate biologic principles into clinically usable state variables, radiotherapy platforms, and biomarker-embedded trial concepts. Clinicians may use the framework to support multidisciplinary discussion, whereas trialists and translational researchers may use it to refine prospective study design.

## 3. Methods: Literature Search and Selection for This Educational Narrative Review

This manuscript is a narrative (non-systematic) educational review. The authors prioritized three categories of evidence: (1) high-quality clinical sources that define current osteosarcoma management and prognostic factors, (2) precision oncology literature characterizing osteosarcoma’s genomic architecture and heterogeneity, and (3) translational radiobiology literature relevant to SBRT/SABR, SFRT (spatially fractionated radiotherapy), immune modulation, and non-targeted effects.

Evidence was selected for **conceptual relevance** to an educational framework rather than for quantitative synthesis or pooled effect estimation. Targeted keyword searches (e.g., osteosarcoma, radiobiology, SBRT/SABR, precision medicine, SFRT) were supplemented by PubMed’s “similar articles” function and citation snowballing to identify additional sources. Because this review is intended to be educational and hypothesis-generating, it is **not** a systematic review, and the references included are **not exhaustive**.

A note about SBRT versus SABR nomenclature used for the purposes of this paper (**terminology clarification): Stereotactic body radiotherapy (SBRT)** is used as an overarching term encompassing stereotactic radiotherapy delivered with modern image guidance, motion management, and quality assurance across a range of hypofractionated dose–fractionation schemes. The term **stereotactic ablative radiotherapy (SABR)** is used more specifically to denote regimens employing **ablative dose intensities**, typically ≥600 cGy per fraction, delivered using SBRT-related techniques and quality assurance practices. This distinction is used for conceptual clarity and does not imply separate technologies, but rather differing biologic and dosimetric intents within stereotactic radiotherapy.

## 4. Review and Discussion

### 4.1. Why Mutation-Centric Precision Oncology Underperforms in Osteosarcoma—A Brief Biology Rationale

Precision oncology has transformed outcomes in several malignancies by matching recurrent driver alterations to targeted therapies, but osteosarcoma has not benefited proportionately from mutation-centric paradigms [11,12,13]. Genome-informed analyses consistently show that osteosarcoma is dominated by extensive somatic copy number alterations and structural rearrangements, while recurrent actionable point mutations are relatively uncommon [12]. This genomic architecture contributes to profound inter- and intratumoral heterogeneity and complicates single-target “drug-to-mutation” matching [12,14].

Recent multi-region whole-genome sequencing demonstrates that chromothripsis remains an active, ongoing process in osteosarcoma, driving genome complexity and clonal evolution [14]. Longitudinal sequencing similarly reveals dynamic evolution during progression, including accumulation of structural variants and shifting copy number profiles [15]. Studies of recurrent disease further show the emergence and dominance of subclonal copy number alterations and treatment-resistant clones [16]. Reviews of precision oncology programs—including MATCH-style frameworks—highlight both the promise and the operational challenges of implementing precision approaches in osteosarcoma, given its rarity, tissue limitations, and genomic complexity [17]. A recent open-access review also summarizes how multi-omics subtyping efforts may eventually support more refined precision strategies aligned with this heterogeneity [18].

Together, these data support a **state-based precision model**, in which strategy selection is aligned to tumor state—burden, tempo, prior response, and resectability—rather than to single molecular events [12,13,14,15,16,17,18].

### 4.2. Where Radiobiotherapy May Fit in Osteosarcoma Care: A State-Based Framework

**Figure 2:**
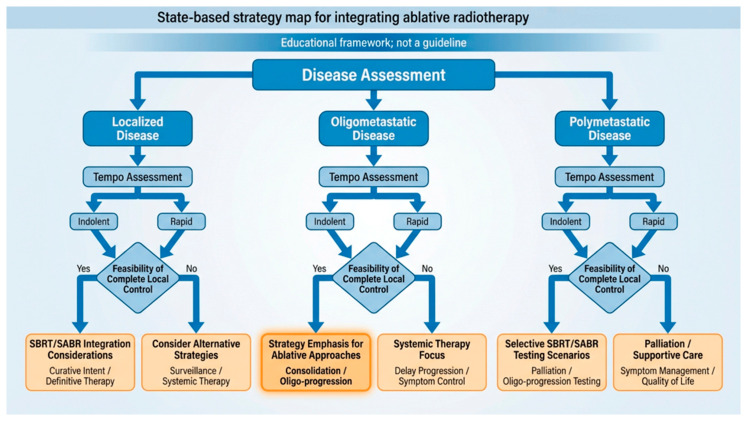
State-based osteosarcoma strategy map. Clinically tractable state variables (burden, tempo, prior systemic response, and feasibility of complete local control) can be used to frame multimodality strategy selection and to identify settings in which ablative radiotherapy (e.g., SBRT/SABR) may be tested as part of biomarker-embedded trials. The figure is intended to support trial design thinking rather than serve as a clinical practice guide.

With this state-based framing in mind, Figure 2 and Table 2 translate these variables into a strategy map and practical phenotype table.

For lung-dominant relapse, complete surgical resection of metastases remains a key predictor of survival; retrospective analyses consistently support an association between pulmonary metastasectomy and improved outcomes in resectable osteosarcoma lung metastases [19]. For unresectable, recurrent, or high-risk lesions, stereotactic body radiotherapy (SBRT) has been explored as a feasible local control modality in metastatic or recurrent osteosarcoma and Ewing sarcoma, though caution is warranted in settings such as re-irradiation and concurrent chemotherapy [20]. Because osteosarcoma has historically been considered relatively radioresistant—and because high-level SBRT evidence remains limited to small series and retrospective reports—SBRT/SABR should be viewed primarily as a **local control tool** for selected lesions [9]. Any systemic or biologic effects should be treated as **secondary, testable hypotheses**, not assumed benefits [21,22].

Osteosarcoma “state” can be operationalized using clinically tractable features such as: (a) localized vs oligometastatic vs polymetastatic burden, (b) tempo of progression, (c) prior chemotherapy response (including necrosis), and (d) feasibility of complete local control [1,7,8].

In relapsed disease, prognosis is strongly influenced by relapse-free interval, metastatic burden, and the ability to achieve complete resection, supporting explicit stratification by these factors in future trials [1,2,3,9,14,15].

**Figure 3:**
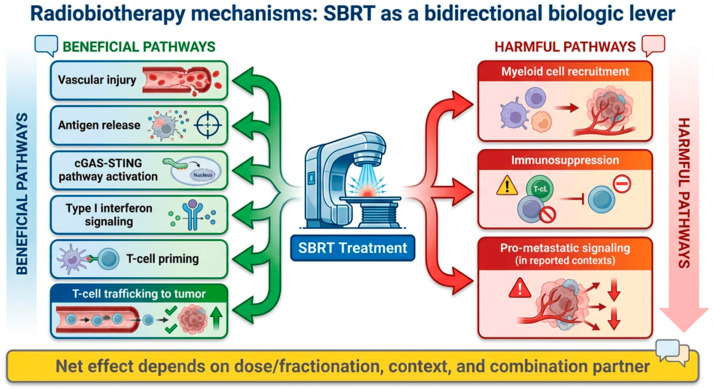
Radiobiotherapy concept—SBRT/SABR as a bidirectional biologic lever. Beyond direct tumor cell kill, ablative radiotherapy can remodel the tumor microenvironment through vascular injury and immune/inflammatory signaling. These effects may support systemic tumor control in some contexts (abscopal responses) but may also promote immunosuppression or pro-metastatic signaling under certain conditions, emphasizing the need for schedule optimization and biomarker-embedded validation.

Considering these state-based “dynamic stages,” Figure 3 and Table 3 translate these variables into a strategy map and practical phenotype table.

### 4.3. Brief Applied Example: Using the State-Based Model in Practice

A practical illustration comes from our recently published case report [9]. The patient developed pulmonary and adrenal metastases with a relatively slow tempo and limited overall burden, and the lesions were considered amenable to definitive local therapy using metastasis-directed approaches (metastasectomy and SABR, as described in the report). Classification within the state-based framework: **oligometastatic lung relapse with slow tempo and potentially fully ablatable disease**.

**Strategy implication:** prioritize complete local control of all visible disease (metastasectomy and/or SABR to all lesions, as feasible) and treat any proposed systemic partner or sequencing choice as a **testable biologic hypothesis**, not an assumed benefit.

In a radiobiotherapy trial context, this state would support:

1. Explicit definitions for “oligometastatic” and “tempo”;
2. Prospective documentation of the feasibility of complete ablation;
3. Embedded correlative sampling (e.g., ctDNA and immune profiling) to evaluate both beneficial and potentially adverse systemic signaling (abscopal vs “badscopal”) rather than relying on anecdotal outcomes.

An interesting aspect of the case was the use of a targeted agent (cabozantinib) combined with preoperative SABR for a recurrent lesion in a challenging anatomic location. Remarkably, post-resection histopathology demonstrated **no viable tumor cells**—an unusual finding in osteosarcoma, which is generally considered radioresistant to conventional radiotherapy [9].

### 4.4. Radiobiotherapy: Definition and Core Concept

Radiobiotherapy describes the deliberate integration of radiotherapy with systemic biotherapies—including cytotoxic chemotherapy, targeted agents, monoclonal antibodies, and immunotherapies—based on biologic rationale rather than solely anatomic targeting [9,10,11,12,13,14,15,16,17,18,19,20,21,22,23]. It is intended as a **framework for mechanism-based combination strategies and trial design**, rather than a clinical practice recommendation [23].

Within this framework, radiotherapy is used as a **purposeful biologic intervention**, providing both high-probability local control and a measurable perturbation of the tumor ecosystem. Combinations are defined by **mechanism, schedule, and embedded biomarkers**, distinguishing radiobiotherapy from routine “concurrent chemoradiation,” where chemotherapy is primarily used as a radiosensitizer without a broader biologic hypothesis.

A conceptual definition of radiobiotherapy is summarized in Table 4. It is intended as an illustrative outline rather than an exhaustive practice management reference.

### 4.5. SBRT Radiobiology: Why Dose and Fractionation Matter

Modern stereotactic techniques enable highly conformal delivery of large fraction sizes to limited volumes, creating radiobiological conditions distinct from conventional fractionation [9,24]. Much of the mechanistic and clinical immunology literature informing these concepts derives from melanoma, non-small cell lung cancer (NSCLC), and other more common solid tumors; ***therefore, the discussion here reflects an extrapolated biologic rationale rather than osteosarcoma-specific proof.***

High-dose stereotactic radiotherapy can induce indirect tumor cell death mechanisms—including vascular injury and immune-associated effects—in addition to direct clonogenic cell kill [24]. A foundational review of vascular biology highlights that doses above ~10 Gy per fraction can cause severe vascular damage in experimental tumors, with implications for SBRT/SRS/SABR and indirect tumor cell death through microenvironmental disruption [24]. A critical perspective on SBRT radiobiology argues that, for many tumors, classic radiobiological principles (the “5 Rs”) may remain sufficient to explain excellent outcomes, while acknowledging that enhanced antitumor immunity may occur in selected contexts [24,25]. SBRT is widely viewed as immunomodulatory, and there is substantial interest in combining SBRT with immunotherapies to augment both local and systemic antitumor responses [9,23].

Radiotherapy (Figure 3) can remodel inflammatory signaling and tumor microenvironment contexture through tumor cell autonomous pathways, including the activation of the cyclic GMP–AMP synthase (cGAS)–stimulator of interferon genes (STING) pathway via micronuclei—an innate immune DNA-sensing mechanism that can drive type I interferon signaling. Extracellular vesicle biology has also been incorporated into modern radiobiology frameworks, including expanded “six Rs” models that explicitly incorporate immune reactivation. Tumor-associated macrophage (TAM) polarization and reprogramming are central to tumor ecosystem behavior and may be influenced by radiotherapy, representing another mechanistic integration point [25,26,27].

Table 5 summarizes key radiobiology mechanisms relevant to SBRT/SABR combinations and highlights why dose and fractionation decisions are biologically meaningful.

From a practical perspective, one question is how commonly used SABR dose/fractionation schedules may differ in their clinical tradeoffs and in their hypothesized immunologic implications. Table 6 provides a simplified overview.

**Abscopal and Non-Targeted Effects: Opportunity, Limits, and New Cautions**

The abscopal effect—regression of distant, non-irradiated tumors following localized radiotherapy—has been linked to immune-mediated mechanisms, but remains uncommon in routine clinical practice [28,29,30,31,32,33,34,35,36]. Mechanistic studies demonstrate that dose and fractionation strongly influence immunogenicity; for example, induction of Trex1 at higher single-fraction doses can attenuate immune activation by degrading cytosolic DNA, underscoring the importance of fractionation when combining radiotherapy with immunotherapies [27,28]. Preclinical evidence further shows that **fractionated regimens**, but not single-dose regimens, may be required to elicit abscopal responses when paired with immune checkpoint blockade [27,28,29].

**Figure 4:**
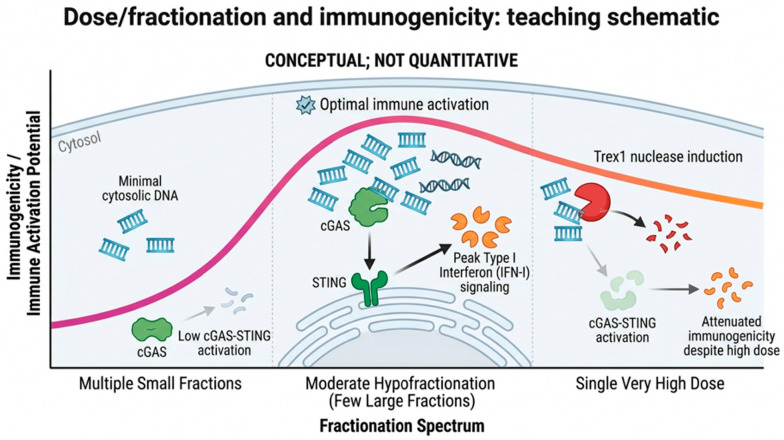
Dose/fractionation and immunogenicity—a conceptual teaching schematic. This figure illustrates—conceptually rather than quantitatively—how different fractionation choices may influence immune activation, including cytosolic DNA signaling and downstream pathways such as cGAS–STING. Moderate hypofractionation is shown as potentially supporting stronger immune activation, whereas very high single-fraction doses may attenuate immunogenicity through mechanisms such as Trex1 induction. The schematic is intended to support trial design thinking about schedule optimization rather than to prescribe a specific regimen.

To illustrate the schedule-dependent nature of immunogenicity—and why fractionation matters—Figure 4 presents a simplified schematic linking dose/fractionation choices to immune activation and potential attenuation pathways.

### 4.6. Abscopal and Non-Targeted Effects: Opportunity, Limits, and New Cautions

Clinical reports document abscopal responses in humans, including early mechanistic correlates in melanoma and a reported abscopal response in treatment refractory NSCLC treated with radiotherapy plus ipilimumab [29,30,34,35]. A landmark study demonstrated that radiation combined with dual checkpoint blockade can activate non-redundant immune mechanisms, informing rational triplet combinations in selected contexts [31]. Reviews emphasize that radiotherapy–immunotherapy combinations may increase abscopal response rates, while also highlighting limitations, variability, and barriers to reproducibility [32,33,34,35]. A comprehensive review further delineates bystander and abscopal effects and mechanisms relevant to radiotherapy–immunotherapy integration [33]. Contemporary clinical perspectives summarize abscopal responses in patients receiving immunotherapy and discuss emerging biomarkers and resistance mechanisms [33,34,35,36].

Importantly, emerging evidence also cautions that radiotherapy can, under certain conditions, promote distant metastatic progression. A 2025 *Nature* study reported that radiotherapy can induce amphiregulin expression in tumor cells, reprogramming EGFR-expressing myeloid cells toward immunosuppression and stimulating distant metastasis growth—sometimes termed a “badscopal” effect [36]. Educationally, this underscores that radiobiotherapy is a **bidirectional biologic lever**, requiring careful trial design, mechanistic counter-strategies, and biomarker evaluation rather than assumptions of net benefit [36,37]. Predicting the balance between beneficial versus adverse immunomodulation, measuring minimal residual disease, and adapting clinical trials to capture these dynamics remain active areas of investigation [38,39,40,41].

### 4.7. Spatially Fractionated Radiation Therapy (SFRT) for Bulky or Unresectable Sarcoma States: Where It May Fit

Spatially fractionated radiation therapy [42,43,44,45,46,47,48,49,50,51] (SFRT) encompasses techniques—historically “GRID” and more recently three-dimensional “lattice” approaches—that intentionally deliver a highly heterogeneous dose distribution within a bulky tumor, creating alternating high-dose “peaks” (vertices) and lower-dose “valleys.” SFRT has re-emerged as a practical option for very large, symptomatic, and/or unresectable tumors where uniform dose escalation is limited by normal-tissue constraints. In contemporary sarcoma series, SFRT is often delivered as an upfront high-dose spatial “boost” followed by conventional or moderately hypofractionated external beam radiotherapy (EBRT), with reported symptom relief and radiographic responses in selected patients [42,43,45].

From a radiobiology perspective, SFRT is frequently framed as a form of partial-volume irradiation that may enable clinically meaningful tumor responses while improving normal-tissue tolerance. Proposed—but still incompletely validated—mechanisms include vertex-centered microvascular disruption and indirect tumor cell death, radiation-induced bystander signaling from high-dose regions into adjacent lower-dose compartments, and immune/inflammatory remodeling that may differ from uniform dose plans. Because SFRT dosimetry is inherently heterogeneous, studies increasingly report technical descriptors such as peak dose, valley dose, vertex size/spacing, and peak-to-valley dose ratio; however, these parameters are not yet standardized across trials [43].

Within this review’s state-based framework, SFRT is most relevant to states characterized by bulky primary or locoregional disease that is unresectable or morbidity-prohibitive, where the dominant objective is symptom relief and/or achieving sufficient tumor regression to enable later surgery or other definitive local therapy. Conceptually, SFRT **complements** SBRT/SABR rather than replaces it: SBRT/SABR typically treats small targets uniformly to ablative doses with steep falloff, whereas SFRT is designed for very large targets by accepting heterogeneity to respect normal-tissue constraints. Bottom line: SFRT is best presented as a **pragmatic bulky disease strategy** and a research opportunity—particularly for biomarker-embedded studies of bystander and immune signaling—rather than a standardized osteosarcoma regimen [43,44,45,46,47,48,49,50,51].

### 4.8. Additional Discussion: SFRT Within a Radiobiotherapy Framework (Bulky Disease, Endpoints, and Open Questions)

While SBRT/SABR is most naturally applied to small-volume targets, SFRT may be particularly useful when sarcoma burden is dominated by bulky and/or unresectable disease and uniform dose escalation would exceed normal-tissue constraints. Across contemporary lattice/GRID experiences, SFRT is commonly used as a spatial boost integrated into a broader EBRT course, with frequent reports of symptom improvement and local responses in selected cohorts (often treated with palliative intent). However, the SFRT literature remains heterogeneous in technique, prescription, and outcome reporting, and prospective comparative data are still emerging. Accordingly, in an osteosarcoma-focused educational review, SFRT is most appropriately framed as a **promising, hypothesis-generating approach** that requires standardized reporting and prospective, biomarker-embedded evaluation [43,44,45,46,47,48,49,50,51].

Mechanistically, SFRT can be viewed as an extreme form of spatial dose heterogeneity: very high-dose vertices embedded within lower-dose tumor “valleys.” Proposed biologic consequences—still incompletely validated—include vertex-centered vascular injury, gradients of inflammatory signaling, and non-targeted/bystander effects that may propagate beyond high-dose regions. These hypotheses intersect with broader radiobiotherapy themes discussed for SBRT/SABR (microenvironment remodeling, innate immune activation, and myeloid reprogramming), but SFRT may create distinct tissue states because large valley volumes remain relatively spared compared with uniform plans [43,47,48,49,51].

SFRT is not simply “dose escalation by another name.” Its clinical and biologic behavior depends on its deliberately heterogeneous geometry and on how the SFRT boost is integrated with any external beam radiotherapy (EBRT) base dose. Across published series, key dosimetric descriptors vary substantially—including peak dose, valley dose, peak-to-valley dose ratio, vertex diameter and spacing, number and placement of vertices, and whether (and when) an EBRT “base dose” is delivered [44]. From a practical standpoint, sarcoma targets may involve skin, subcutaneous tissues, bone, neurovascular bundles, or postoperative beds; therefore, toxicity considerations include skin breakdown, wound-healing complications (particularly when combined with preoperative radiotherapy), neuropathy, pathologic fracture risk, and cumulative dose constraints if retreatment is contemplated [42,43,45,48,49,50].

For osteosarcoma specifically, the most plausible SFRT use-cases align with state-based categories in which the dominant challenge is **bulky local disease**—for example, axial or pelvic primaries, locally recurrent tumors, or large soft tissue components that are unresectable at presentation or require downstaging to make surgery feasible [10,42,43,45]. In these settings, SFRT can be conceptualized as either:

1. **Palliation**, with an emphasis on rapid symptom relief;
2. A **“bridge-to-definitive-local-therapy”** strategy in which early volumetric response facilitates later resection or reconstruction [42,43,44,45].

To maintain scientific rigor in this educational review, the most important trial design consideration is **endpoint selection**. Beyond RECIST, relevant measures include patient-reported pain and function trajectories, time to local progression, conversion-to-resectability rates, wound-healing and fracture outcomes, and integration of systemic biomarkers such as circulating tumor DNA (ctDNA) as a measure of residual disease burden, and immune/myeloid signatures as indicators of systemic perturbation [10,39,40,41,42,43,44,45,51].

### 4.9. Key Educational Research Questions for SFRT (Especially in Rare Tumors Such as Osteosarcoma)

At this time, several questions must be addressed in clinical research designs:

- Which patient/tumor “states” benefit most? This includes clarifying whether SFRT is most effective for bulky symptomatic palliation, conversion-to-resectability, or local recurrence scenarios [10,42,43,44,45].
- What SFRT parameters should be standardized and reported? Key dosimetric descriptors—such as peak and valley doses, peak-to-valley ratio, vertex geometry, and EBRT base dose integration—require harmonization to enable comparison across trials [43,48,49,50].
- How should SFRT be sequenced with surgery and systemic therapy? Optimal sequencing is essential to maximize benefit while minimizing wound-healing complications, skin toxicity, and bone-related risks [19,42,45,50].
- What biomarkers best reflect response and risk, and how should they be sampled? Potential candidates include symptom endpoints, volumetric response, ctDNA dynamics, and immune/myeloid signatures, each requiring thoughtful timing and integration [26,39,40,41,42,45,51].

### 4.10. Overall Implications for Clinical Trial Design in Osteosarcoma

Clinical trials in osteosarcoma face persistent challenges, including **rarity**, **biologic heterogeneity**, and **endpoints that may not capture the benefits of integrated local-systemic strategies** [3,4,12,16,17,40,41]. Precision radiation oncology initiatives emphasize that genomic and biologic metrics increasingly influence radiation personalization and combination logic [11,12,37]. Systems pharmacology modeling has also been applied to analyze radiation plus PD(L)1 combinations and to prospectively evaluate sequences and schedules, supporting **in silico optimization** approaches [38].

**Figure 5:**
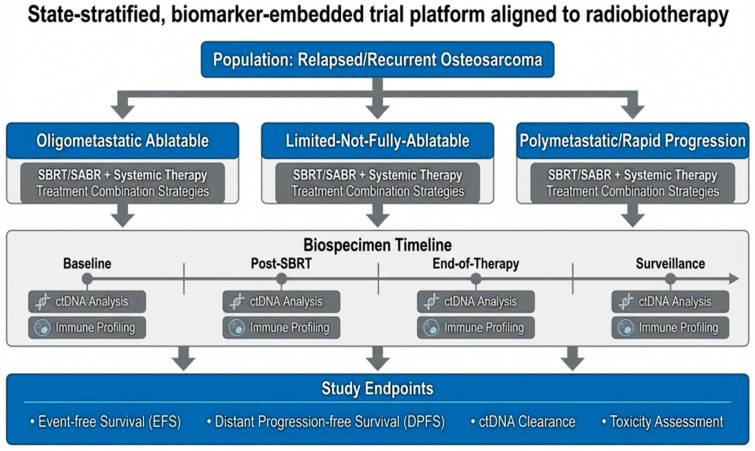
Example trial architecture aligned to a radiobiotherapy framework. A state-stratified platform can test the integration of SBRT/SABR with systemic partners while embedding serial biomarkers (e.g., ctDNA minimal residual disease dynamics and immune profiling) to identify which disease states derive benefit and to detect potentially adverse systemic signaling.

An example of platform-style trial schematic aligned with this approach is shown in Figure 5.

Table 7 [39,40,41,42,52,53,54,55,56,57,58,59] provides example trial concepts that operationalize this framework using biomarker-embedded, state-stratified designs in oligometastatic disease-states intended to accelerate learning in a rare and heterogeneous disease.

Because immune effects depend on dose and fractionation, schedule optimization is integral to radiobiotherapy trial design [21,27,28,38]. Adaptive enrichment designs have been increasingly utilized to balance all-comers’ enrollment with biomarker-defined subpopulations, potentially improving efficiency in heterogeneous diseases [12,40,41]. Biomarkers for minimal residual disease (MRD), such as circulating tumor DNA (ctDNA), have been proposed as potentially transformative for detecting residual disease and predicting relapse after definitive therapy, though implementation requires careful validation [39,54,59]. Foundational work in adaptive trials supports the broader rationale for adaptive, learning-oriented strategies [40,41].

**Figure 6:**
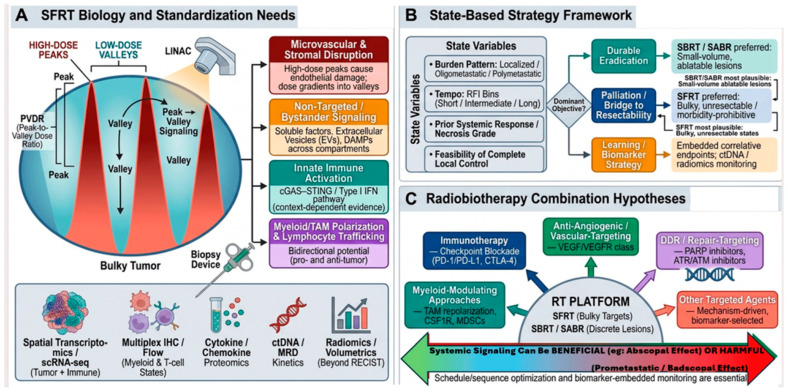
Integrating SFRT biology (including multi-omics), state-based precision strategy selection, and radiotiotherapy combination logic: a conceptual framework. Panel (**A**) shows SFRT peak–valley dose geometry (LINAC) and key mechanisms: microvascular/stromal disruption, non-targeted/bystander signaling, cGAS–STING/type I IFN innate activation, and myeloid/TAM polarization with lymphocyte trafficking. Correlative readouts include spatial transcriptomics/single-cell RNA-seq, multiplex IHC/flow cytometry, cytokine/chemokine proteomics, ctDNA/MRD kinetics, and radiomics/volumetrics. Panel (**B**) outlines a state-based strategy in which burden pattern, tempo (recurrence-free interval [RFI] bins), prior systemic response/necrosis grade, and feasibility of complete local control determine the dominant objective: durable eradication (favor SBRT/SABR), palliation/bridge-to-resectability for bulky unresectable disease (favor SFRT), or a learning/biomarker strategy with embedded correlatives. Panel (**C**) summarizes radiobiotherapy combinations: SFRT and SBRT/SABR as radiation platforms paired with checkpoint blockade, VEGF/VEGFR-class vascular targeting, DDR/repair targeting (e.g., PARP, ATR/ATM), and myeloid-modulating approaches (e.g., TAM repolarization, CSF1R, MDSC strategies). Bidirectional systemic signaling—beneficial (abscopal) or harmful (pro-metastatic/“badscopal”)—supports schedule/sequence optimization and biomarker monitoring. Abbreviations: PVDR, peak-to-valley dose ratio; SFRT, spatially fractionated radiotherapy; SBRT, stereotactic body radiotherapy; SABR, stereotactic ablative body radiotherapy; TAM, tumor-associated macrophage; DDR, DNA damage response; ctDNA, circulating tumor DNA; MRD, minimal residual disease; RFI, recurrence-free interval; IFN, interferon; DAMPs, damage-associated molecular patterns; EVs, extracellular vesicles; LINAC, linear accelerator; MDSCs, myeloid-derived suppressor cells.

Taken together, the SFRT discussion above and the state-stratified trial concepts in Table 7 motivate a single integrative idea for translational programs: treat spatial dose heterogeneity (SFRT/GRID/lattice) and ablative uniform dose (SBRT/SABR) as complementary radiation “platforms,” then match each platform to the patient’s state (burden, tempo, and feasibility of complete local control) and pair it with mechanism-based systemic partners while measuring multi-omics and biomarker readouts prospectively. Figure 6 provides a conceptual schematic intended to help trainees and trial designers visualize how SFRT biology (including modern multi-omics), state-based strategy selection, and radiobiotherapy combination logic can be connected in a hypothesis-generating manner.

A second educational takeaway is that radiotherapy may be viewed not only as a local control modality but also as a dose-, fractionation-, and context-dependent biologic intervention whose systemic consequences require prospective validation.

## 5. Limitations

Evidence supporting radiobiotherapy concepts in osteosarcoma remains limited and is largely extrapolated from mechanistic radiobiology and broader oncology experiences, including melanoma, NSCLC, and other solid tumors [21,27,28,29,30,31,32,33,34,35,36,37,38]. Much of the SBRT/SABR radiobiology and immuno-oncology literature is therefore not osteosarcoma-specific, in part because osteosarcoma has historically been considered relatively radioresistant and radiotherapy has played a more restricted role in standard management [1,3,4,9,20,21]. Similarly, the emerging SFRT (GRID/lattice) literature is predominantly sarcoma-general, often includes mixed histologies, and remains characterized by heterogeneous techniques and early phase outcome reporting, such that osteosarcoma-specific evidence is still sparse [42,43,44,45,49,50,51].

Recent advances in osteosarcoma biomarker and liquid biopsy research may help refine future state-based clinical trials by improving risk stratification, minimal residual disease assessment, and longitudinal monitoring. In particular, circulating tumor DNA (ctDNA) is emerging as a promising investigational tool for relapse prediction and dynamic response assessment, although osteosarcoma-specific validation remains limited and technical challenges related to structural variation, assay standardization, and low disease burden persist. Broader liquid biopsy approaches, including circulating tumor cells, extracellular vesicles, and integrated blood-based correlatives, may eventually provide complementary biologic information, but these applications remain early and should currently be regarded as hypothesis-generating rather than trial-defining [11,12,13,14,15,16,25,39,54,59,60,61,62,63,64,65,66,67,68]. These concepts are detailed in Table 8.

Additional barriers also deserve emphasis. A related biologic hypothesis worth testing is that carefully selected SABR/SBRT schedules may enhance tumor antigen release, innate immune sensing, and subsequent immune visibility in osteosarcoma, thereby partially countering its frequently immunologically constrained or “immune-cold” microenvironment; however, this concept remains extrapolated largely from broader radiobiology and immunogenic-cell-death literature, together with limited osteosarcoma-specific clinical observation, and should not be interpreted as an established therapeutic effect [9,21,24,27,28,30,65,66,67]. Furthermore, prospective trial development in osteosarcoma is inherently difficult because of disease rarity, biologic heterogeneity, small eligible populations within specific state-defined subsets, and the practical burden of multi-institutional biomarker collection [3,4,12,16,17,40,41,45,68]. Cumulative toxicity also remains a major concern, particularly in patients undergoing multimodality therapy, re-irradiation, surgery, reconstruction, or treatment near weight-bearing bone and critical normal tissues. Finally, although ctDNA and other liquid biopsy approaches are promising, biomarker reproducibility remains limited by assay heterogeneity, low disease burden, variable shedding, timing of sample acquisition, and incomplete standardization across platforms [25,39,48,53,61]. These factors reinforce the need for cautious interpretation and carefully staged prospective validation [40,41,43,68].

Taken together, the limited osteosarcoma-specific clinical evidence and the still-evolving biomarker landscape mean that the concepts outlined here should be interpreted as tools for prospective study design rather than definitive clinical guidance. Future progress will depend on improved trial structure, feasible biomarker standardization, reproducible sampling strategies, and clear separation of exploratory correlatives from validated clinical decision tools.

## 6. Summary

Osteosarcoma illustrates a broader challenge in modern oncology: biology and clinical behavior do not always map cleanly onto static anatomic stage or single-gene targets. This review synthesizes evidence that osteosarcoma’s copy-number/structural-variant-driven genomic architecture and heterogeneity can limit mutation-centric precision strategies, motivating a complementary **state-based approach** that emphasizes disease burden pattern, tempo, prior treatment response, and feasibility of complete local control.

Across cancers, an expanding radiobiology literature supports viewing radiotherapy—especially SBRT/SABR and spatially fractionated approaches—as more than a local cytotoxic modality. Dose, fractionation, and spatial distribution can reshape tumor vasculature, inflammation, innate DNA sensing (e.g., cGAS–STING), and myeloid/lymphoid programs, creating a plausible mechanistic basis for combination therapy. What remains to be defined, in osteosarcoma and beyond, is how to reliably translate these **bidirectional** effects into net clinical benefit.

Key unknowns include: which tumor/host states are most “actionable” by local-systemic integration; which radiation schedules and platforms best match specific biologic hypotheses; which biomarkers (ctDNA/MRD kinetics, immune/myeloid signatures, cytokine panels, radiomics/volumetrics, patient-reported outcomes) are feasible, reproducible, and predictive; and how to detect and mitigate unintended systemic consequences (including rare abscopal benefit and potential pro-metastatic signaling).

In this sense, radiobiotherapy is best regarded as an **investigational, cross-disease framework** for designing biomarker-embedded studies that treat radiation parameters as biologic variables—analogous to dose and schedule in systemic therapy—rather than as fixed technical details.

### 6.1. Relationship to Prior Work and Avoiding Duplicate Publication

The lead authors have recently published a case report describing a single patient’s clinical course and management that contributed to the conceptual development summarized in this review [9]. They also plan to submit a more translational, research-oriented perspective paper. These manuscripts are distinct in aims, scope, and content: the current article is a **narrative educational review** that synthesizes published clinical and translational literature to propose a generalizable, state-based conceptual framework for integrating ablative radiotherapy into osteosarcoma strategies.

This article does **not** reproduce patient-level text, images, tables, timelines, or unique clinical details from the case report. Any overlap is limited to brief, high-level background statements that are standard in the field and rewritten for the educational review context.

### 6.2. Key Takeaways

- Osteosarcoma biology and genomic architecture often limit mutation-centric “drug-to-mutation” precision approaches; strategy selection may be more effective when aligned to **tumor state** (burden, tempo, chemosensitivity, and feasibility of complete local control). SBRT/SABR can provide high local control for selected metastatic/recurrent lesions and may also act as a **biologic modifier** via vascular injury and immune/inflammatory signaling. **Dose and fractionation matter** for immunogenicity; schedule optimization is part of combination-therapy design, not a technical afterthought. Abscopal responses are real but uncommon; radiotherapy can also plausibly promote distant progression in certain biologic contexts, underscoring the need for **biomarker-embedded trials**. Radiobiotherapy should be treated as a **testable framework** requiring prospective validation, not as routine care guidance. Appendix A and Appendix B further expand on these ideas [11,12,13,14,15,16,25,39,54,59,60,61,62,63,64,65,66,67,68,69,70,71,72].

## 7. Conclusions

Modern radiobiology indicates that high-dose stereotactic radiotherapy can exert vascular, inflammatory, and immune-modulating effects relevant to combination therapy; however, these effects are **bidirectional and context-dependent**. For osteosarcoma—where rarity and heterogeneity complicate trial design—the next step is to convert this concept into measurable, prospective programs.

For **trainees and learners** (students, residents, fellows), a practical action item is to adopt a shared vocabulary: state variables (burden, tempo/RFI, prior response, ablatability), radiation “platforms” (SBRT/SABR vs. SFRT), and biologic hypotheses (cGAS–STING, myeloid/TAM reprogramming, bystander/non-targeted effects). For each case, the key question becomes: **what should be measured, and when?** (ctDNA, immune/myeloid signatures, cytokines, patient-reported outcomes).

For **clinical oncologists** (surgical, radiation, and medical), a near-term action is to strengthen multidisciplinary state-based decision-making by explicitly documenting feasibility of complete local control, normal-tissue constraints, and the intended objective (eradication vs palliation vs bridge-to-resectability), and by preferentially routing eligible patients to prospective protocols.

For **laboratory and translational researchers**, priority actions include developing assays and models that reflect osteosarcoma’s structural-variant- and copy-number-driven heterogeneity; testing dose/fractionation and spatial heterogeneity as biologic variables; and pairing radiation exposure with mechanistic readouts (myeloid and T-cell states, interferon programs, extracellular vesicles, ctDNA kinetics) to identify both beneficial and potentially pro-metastatic signaling.

For **clinical trialists**, the actionable agenda is to build **state-stratified, biomarker-embedded trial platforms** that:

1. Prespecify lesion treatability and local control feasibility;
2. Explicitly test dose/fractionation and sequencing rather than treating them as fixed;
3. Use endpoints beyond short-term RECIST response (e.g., ctDNA clearance, time to new metastases, symptom/function trajectories, conversion-to-resectability);
4. Monitor for bidirectional systemic effects (abscopal and “badscopal”) with predefined safety and stopping rules.

**The central take-home:** SBRT/SABR should be viewed not only as a local control tool, but as a **hypothesis-generating biologic lever** whose optimal integration in osteosarcoma must be defined prospectively through coordinated clinical, translational, and trial design efforts.

More broadly, the rarity of osteosarcoma should not be viewed only as a barrier, but also as a reason for stronger cooperative clinical trial organization. Pediatric oncology has shown through highly collaborative group-based research in rare tumors such as Wilms tumor and other childhood cancers that meaningful progress is possible when large proportions of eligible patients are enrolled in shared prospective studies. Because osteosarcoma spans children, adolescents, and young adults, future progress will likely depend on similarly coordinated, multi-institutional, state-stratified, biomarker-embedded trial platforms that can convert biologic hypotheses into reproducible clinical knowledge.

## 8. Future Directions

Future work should investigate whether the concept of state-based decision-making can inform osteosarcoma care more broadly, beyond radiotherapy alone. In principle, this framework may help organize multidisciplinary cancer care decision-making by integrating disease burden, tempo of progression, prior treatment response, feasibility of complete local control, and evolving biologic information. However, this concept remains educational and hypothesis-generating at present, and its broader role in cancer care decision-making must be evaluated carefully in prospective clinical and translational studies.

A major next step will be the development of state-stratified, biomarker-embedded clinical trials that test not only radiotherapy platforms and dose/fractionation strategies, but also how state-based logic may guide patient selection, endpoint choice, and adaptive treatment planning across different osteosarcoma scenarios. The suggestions outlined in this review are intended to support that next phase of investigation. More broadly, if validated, this framework could contribute to a more patient-focused precision cancer care model in osteosarcoma—one that aligns treatment decisions not only with tumor biology, but also with disease behavior, feasibility of local control, and the individual clinical context.

## Figures and Tables

**Table 1:** Stage-based versus state-based frameworks in osteosarcoma. This table compares how conventional staging and a state-based approach differ in their core questions, typical inputs, decision-making implications, and potential use in strategy selection and clinical trial design.

Feature	Stage-Based Framework	State-Based Framework (Used in This Review)
Core question	“Where is the cancer (anatomic extent) right now?”	“What is the cancer doing right now, and what is feasible to control it?”
What it is	An anatomic classification of disease extent at a point in time (e.g., localized vs metastatic).	A clinically actionable description of the disease’s current behavior and constraints that can change over time.
Typical inputs	Primary tumor extent/size, presence of distant metastasis (and sometimes grade). Nodal status is often less central in osteosarcoma.	Burden pattern (localized/oligometastatic/polymetastatic), tempo of progression, prior chemosensitivity (e.g., necrosis response), resectability/ablatability, patient constraints (age, prior RT/surgery, organ reserve).
How it guides decisions	Broad prognosis grouping and standard treatment pathways (e.g., localized: surgery + chemotherapy; metastatic: intensified multimodality approaches).	Strategy selection within and across stages (e.g., attempt complete ablation of all known disease vs systemic backbone focus) and trial stratification aligned to feasibility of local control and disease tempo.
Examples in osteosarcoma	“Localized disease at diagnosis” vs. “metastatic at diagnosis.”	“Oligometastatic lung relapse with all lesions ablatable” vs. “polymetastatic rapid progression despite chemotherapy.”
Best use	Initial risk communication, baseline treatment planning, and eligibility definitions.	Relapse/metastatic strategy selection, integration of local therapies (metastasectomy/SBRT/SABR), and hypothesis-driven trial design.
Key limitation	May not capture evolution, prior treatment response, tempo, or feasibility of complete local control—factors that often drive outcomes in relapse settings.	Not a replacement for staging; it is an overlay that must be operationalized with clear definitions to avoid ambiguity.

**Table 2:** State-based osteosarcoma phenotypes and potential roles for ablative radiotherapy.

Tumor State (Simplified)	Dominant Objective	Typical Local Control Options	Where SBRT/Ablative RT (SABR) May Fit (Hypothesis-Generating)	Key Cautions/Research Needs
Localized, resectable primary	Durable local control + systemic micrometastatic control	Surgery ± perioperative chemotherapy	Generally limited role; potential research role in margin-challenged sites or unresectable subsets (axial and pelvic osteosarcomas)	Long-term toxicity in young patients; need prospective indication definition
Localized but unresectable or morbidity-prohibitive	Local control/palliation/conversion-to-resectability/prevent distant metastases	Radiotherapy, systemic therapy, selected surgery	For bulky targets, consider SFRT (GRID/lattice) as a high-dose spatial boost followed by EBRT; SBRT/ablative RT may be used for selected subvolumes or as consolidation in smaller residual lesions	Bone/soft tissue toxicity; fracture risk; integration with reconstruction
Oligometastatic relapse (lung- or bone-dominant), potentially ablatable	Complete eradication of visible disease	Metastasectomy, ablation, (SBRT– SABR)	SBRT as alternative/complement when surgery not feasible; multi-site ablation strategies	Patient selection; cumulative dose constraints; endpoints beyond RECIST
Polymetastatic or rapidly progressive disease	Symptom control; systemic disease modulation	Systemic therapy ± palliative radiotherapy/selected metastases SABR	Selective SBRT for dominant lesions (pain, bleeding, impending fracture) or for trial-based immunomodulation	Risk of overtreatment; need biomarkers to identify who benefits
Chemotherapy-nonresponsive or poor necrosis responders	Learning/biomarker strategy; local control	Surgery; trial systemic options	SBRT as biologic “lever” in biomarker-embedded trials; evaluate immune and ctDNA dynamics	Avoid assuming immunogenic benefit; monitor potential pro-metastatic signaling

Abbreviations: RT, radiotherapy; SBRT, stereotactic body radiotherapy; SABR, stereotactic ablative radiotherapy; RECIST, response evaluation criteria in solid tumors.

**Table 3:** Suggested operational definitions for state-based variables (illustrative; not consensus criteria).

State Variable	Illustrative Operationalization (Examples)	Why This Matters
Burden pattern: oligometastatic vs. polymetastatic	Oligometastatic: limited number of metastatic lesions that are all amenable to complete local therapy (surgery and/or ablation and/or SBRT/SABR). In many solid tumor trials, “limited” commonly means ~1–5 lesions (sometimes also limiting the number of involved organs); osteosarcoma-specific consensus thresholds are not established.	Trial eligibility and stratification depend on explicit lesion-count and “all sites treatable” criteria.
Tempo/progression kinetics	Tempo can be approximated by relapse-free interval (RFI) and/or imaging growth kinetics. Example bins for educational use: early relapse (RFI < 6 months), intermediate (6–12 months), late (>12 months). Alternative trial cutpoints (e.g., 3 or 24 months) may be used depending on question.	RFI is a reproducible clinical proxy for tumor aggressiveness and is strongly prognostic in relapsed osteosarcoma.
Feasibility of complete local control	“Ablatable” means all known sites can be treated to definitive intent within normal-tissue constraints (by metastasectomy, ablation, and/or SBRT/SABR), with acceptable anticipated morbidity. This should be defined prospectively (e.g., by organ-at-risk constraints and surgical criteria).	Complete resection/ablation is one of the strongest predictors of survival in relapsed disease, so it is a key state variable.

Abbreviations: SBRT, stereotactic body radiotherapy; SABR, stereotactic ablative radiotherapy; RFI, relapse-free interval.

**Table 4:** What is radiobiotherapy and what is not (a few examples).

Defining Principles	What Is It?	What Is It Not?
State-based (time-varying) logic	A mechanism-driven integration of radiation with systemic biotherapies that is tailored to the patient’s current “state” (e.g., oligometastatic relapse with slow tempo and all sites ablatable) and paired with a plan to measure biologic effects (biomarkers/correlatives).	A stage-only cookbook (e.g., “metastatic = give RT + drug X”) without explicit state definition, mechanism, schedule/sequencing, and biomarker plan.
Radiation + a systemic partner (broadly defined)	The partner may be cytotoxic chemotherapy, targeted therapy, monoclonal antibodies, immunotherapy, or combinations—selected because a testable interaction with radiation biology is hypothesized (including dose, fractionation, and sequencing).	Generic “concurrent chemoradiation” where the systemic agent is used primarily as a radiosensitizer without a broader biologic hypothesis or correlative plan (a common paradigm in other cancers).
Radiation as a biologic lever (local + systemic signaling)	Radiation is used intentionally for high-probability local control and for biologic perturbation of the tumor ecosystem (vascular injury, inflammatory/immune remodeling). Effects may be beneficial (abscopal) or harmful (“badscopal”), so trials should prospectively measure both.	A promise that abscopal benefit is expected or routine; or use of radiation as a purely technical local modality with no biologic intent or measurement.
Integration across radiation medicine modalities	When relevant, the framework can encompass external beam RT (including SBRT/SABR), brachytherapy, and radiopharmaceutical/radiotheranostic approaches as “radiation delivery platforms,” each paired with mechanism-based systemic partners and state-defined objectives.	A claim that all radiopharmaceutical or theranostic advances are automatically “radiobiotherapy”; inclusion should be justified by a concrete biologic hypothesis and measurable endpoints.
Radiation as another “drug” inducing systemic effects (in combination with systemic agents) *	Radiation is treated as a doseable, schedule-dependent therapeutic with on-target (local/regional control) and off-target (systemic signaling) effects that can be intentionally paired with systemic agents (e.g., immunotherapy, targeted agents, myeloid/vascular modulators) to test mechanistic synergy. This includes using different radiation delivery platforms (SBRT/SABR for small-volume lesions; SFRT for bulky disease) to create distinct biologic perturbations that are measured with embedded biomarkers.	An assumption that “more radiation” automatically improves systemic outcomes, or that systemic benefit is guaranteed. Also, not a justification for empiric RT + drug combinations without explicit mechanism, sequencing rationale, normal-tissue constraints, and prospective monitoring for both desirable (abscopal) and undesirable (pro-metastatic/immunosuppressive) systemic effects.

Abbreviations: SBRT, stereotactic body radiotherapy; SABR, stereotactic ablative radiotherapy; SFRT, spatially fractionated radiotherapy. * Note: ‘radiation as another “drug” concept’ is still a hypothesis-generating idea requiring further evidence in osteosarcoma.

**Table 5:** Radiobiology concepts relevant to SBRT combinations.

Concept	What It Means	Why it Matters for Combinations	Practical Implication for Trials
Vascular injury/indirect tumor cell death	High-dose fractions can damage tumor vasculature and microenvironment.	May synergize with agents that exploit hypoxia/vascular disruption or immune recruitment.	Capture microenvironment biomarkers; monitor normal-tissue constraints.
cGAS–STING (cyclic GMP– AMP synthase–stimulator of interferon genes) and inflammatory signaling	Radiation-induced DNA damage can trigger innate immune signaling (DNA sensing via the cGAS–STING pathway), which can promote type I interferon responses.	May support checkpoint blockade synergy, but can be schedule-dependent.	Predefine dose/fractionation; collect immune correlates.
Trex1 induction at very high single doses	Very high single-fraction doses may blunt cytosolic DNA signaling by degrading DNA.	Could reduce immunogenicity for some schedules.	Compare fractionated vs. single-fraction regimens in translational substudies.
TAM polarization/myeloid reprogramming	Radiotherapy can alter macrophage phenotypes toward pro- or antitumor states.	Myeloid biology may determine whether RT helps or harms systemically.	Include myeloid/chemokine panels; consider myeloid-targeted partners.
Bystander/non-targeted effects	Signals from irradiated tissue can affect non-irradiated sites.	Explains both “abscopal” benefit and potential systemic harm.	Build biology monitoring for unintended systemic effects.

Abbreviations: SBRT, stereotactic body radiotherapy; TAM, tumor-associated macrophage.

**Table 6:** Common SABR/SBRT dose–fractionation patterns (illustrative) and their implications.

**Illustrative Regimen Pattern**	**Where It Is Often Used (General Oncology Practice)**		**Practical Considerations** **(Tradeoffs)**		**Hypothesized Biologic/Immune Implications (Conceptual)**
Selected vertebra(e), axial or pelvic bone Single-fraction metastases; some high dose (e.g., lung/liver protocols in 1 × 16–24 Gy) selected settings		Convenient; may increase risk in organs-at-risk depending on site; may be less forgiving for setup/motion		Very high single-fraction doses may induce exonucleases (e.g., Trex1) that could attenuate cytosolic DNA-driven innate signaling in some models; schedule may matter for synergy	
Hypofractionated high dose (e.g., 3 × 8–12 Gy)	Common across lung/liver/adrenal oligometastases; some bone lesions		Balances convenience with normal-tissue constraints; often used for lesions near critical structures		Fractionation may better support immune-mediated mechanisms in some preclinical models; useful backbone for combination trials
Moderate Central lung lesions; Lower per-fraction dose may May provide sustained microenvironmental remodeling; Hypofractionation near-hilar/mediastinal reduce toxicity in sensitive sites; immune effects likely context-dependent and should be (e.g., 5 × 6–10 Gy) regions; sites with tighter may trade some ablative biomarker-measured constraints intensity for safety					
More fractions (e.g., 8–10 fractions, SBRT-like)	Re-irradiation scenarios; larger targets; proximity to serial organs		Primarily selected to meet dose constraints and reduce late toxicity; longer overall treatment time		Biologic effects may differ from classic SBRT; combination rationale may depend more on local control and microenvironment modulation than on strong systemic immune priming
Notes: Regimens are illustrative (site- and protocol-dependent) and not osteosarcoma-specific recommendations. For osteosarcoma, optimal schedules for local control and any systemic immunomodulation remain to be prospectively defined.					

Abbreviations: SABR, stereotactic ablative radiotherapy; SBRT, stereotactic body radiotherapy.

**Table 7:** Biomarker-embedded trial concepts aligned to a radiobiotherapy framework in osteosarcoma.

Trial Concept	Population/State	Intervention Idea	Biomarker/Learning Objective	Endpoint Suggestions
State-stratified local ablation platform	Oligometastatic relapse; limited lesions	Surgery and/or SBRT/SABR to all sites ± systemic partner	ctDNA kinetics as MRD; immune profiling pre/post-radiotherapy	Event-free survival; time to new metastases; ctDNA clearance
Schedule optimization translational substudy	Metastatic/recurrent candidates for SBRT/SABR + immunotherapy	Compare fractionation schedules	Immune signatures; Trex1-related hypotheses; T-cell exhaustion markers	Response durability; systemic lesion control; toxicity
Myeloid-focused combination	Relapsed disease with myeloid-dominant signatures	SBRT/SABR + myeloid-modulating agent	Myeloid reprogramming markers; cytokine panels	Distant progression-free survival; safety
Adaptive enrichment design	Mixed states (predefined strata)	Drop/expand cohorts based on early biomarker response	Identify responder states rather than single-gene responders	Bayesian/adaptive endpoints; learning-focused outputs

Abbreviations: ctDNA, circulating tumor DNA; MRD, minimal residual disease; SBRT, stereotactic body radiotherapy; SABR, stereotactic ablative radiotherapy.

**Table 8:** Emerging biomarker-embedded concepts that may help operationalize state-based clinical trials in osteosarcoma.

Biomarker/Approach	Potential Relevance to State-Based Trials	Current Support in Osteosarcoma	Key Limitations/Caution
Tumor-informed ctDNA	May improve minimal residual disease detection, relapse risk stratification, and dynamic assessment of treatment effect after local or multimodality therapy.	Emerging osteosarcoma-specific evidence suggests postoperative ctDNA positivity is associated with inferior outcomes and may precede radiographic relapse.	Assay design is challenging because osteosarcoma is dominated by structural variation and copy number complexity rather than recurrent shared point mutations.
Serial ctDNA monitoring	May help define disease tempo, detect occult progression earlier than imaging in selected patients, and support adaptive trial enrichment.	Early data are promising but remain limited and not yet standardized across platforms or disease states.	Timing, assay sensitivity, and interpretation at low disease burden remain unresolved.
Circulating tumor cells (CTCs)	Could provide complementary information regarding dissemination biology and metastatic risk in selected translational studies.	Investigational; less mature than ctDNA in osteosarcoma.	Technical reproducibility, rarity of detectable events, and uncertain added value limit near-term clinical integration.
Extracellular vesicles/exosomal cargo	May capture tumor microenvironment signaling, including pathways relevant to treatment response and systemic perturbation.	Conceptually promising and increasingly studied in osteosarcoma biomarker research, but still exploratory.	Lack of standardization, variable isolation methods, and uncertain clinical validity currently limit application.
Immune/myeloid blood correlatives	May help test whether specific radiotherapy schedules produce measurable systemic immune or myeloid changes in biomarker-embedded trials.	Supported mainly by broader radiobiology and immunotherapy literature rather than osteosarcoma-specific prospective trials.	Peripheral blood signatures may not reliably reflect intratumoral biology and are vulnerable to sampling and timing effects.
Integrated multi-analyte longitudinal monitoring	Combining ctDNA, immune correlatives, imaging, and clinical state variables may better support state refinement over time.	Conceptually strong and aligned with adaptive trial design, but prospective osteosarcoma-specific implementation remains limited.	Complexity, cost, assay harmonization, and feasibility in a rare cancer remain substantial barriers.

Abbreviations: ctDNA, circulating tumor DNA; CTCs, circulating tumor cells; MRD, minimal residual disease. Notes: this table is intended as a concise, hypothesis-generating summary of emerging osteosarcoma biomarker and liquid biopsy concepts relevant to future state-based trial design. Current support is strongest for ctDNA/MRD approaches, whereas CTCs, extracellular vesicles, and integrated multi-analyte monitoring remain earlier in development [11,12,13,14,15,16,25,26,39,40,41,48,53,61,65,66,67].

## Data Availability

No new data were created or analyzed in this study. Data sharing is not applicable to this article.

## Appendix A

A concise supplementary summary of selected high-dose radiotherapy concepts worth prospective testing in osteosarcoma is provided in Appendix A.

This appendix is intended as a focused, hypothesis-generating summary of why selected high-dose radiotherapy strategies may be worth prospective testing in osteosarcoma, including immunomodulations induced by SBRT/SABR. Current evidence is restricted to common disease such as cancers of the breast, lung, colon, prostate etc. It is deliberately restrained and emphasizes that current support is limited, largely retrospective, and insufficient for practice-changing conclusions. The purpose is to justify future biomarker-embedded phase I/II studies rather than to imply established efficacy [21,22,24,27,28,29,30,31,32,33,34,35,36,37,38,46,47,48,49,50,51,52,53,58,59,60,61,69,70,71,72].

**Concept Worth Testing**	**Why It Is Biologically/Clinically Relevant**	**Current Support in Osteosarcoma**	**What Future Studies Should Test**
Metastasis-directed SBRT/SABR for limited recurrent disease	Some relapsed patients have limited-volume, potentially ablatable disease in which durable local control may alter progression patterns or delay further dissemination.	Support remains limited to retrospective series, mixed sarcoma cohorts, and case-level observations, but these suggest feasibility and local control in selected lesions.	Prospective state-defined studies should evaluate lesion control, time to new metastases, ctDNA kinetics, and patient selection factors such as relapse-free interval and complete ablation feasibility.
High-dose radiotherapy in chemo-nonresponsive or poor necrosis states	Patients with poor response to systemic therapy represent a biologically high-risk group in whom local strategies may also serve as biologic probes in translational trials.	Evidence is preliminary and hypothesis-generating, including the authors’ recent case experience and limited published reports.	Early phase trials should test whether carefully selected high-dose regimens can improve local control and provide measurable biologic readouts without overstating systemic benefit.
SBRT/SABR as a biologically informative intervention	Radiotherapy dose and fractionation can influence vascular injury, inflammatory signaling, innate immune pathways, and myeloid programs, making schedule a potentially important trial variable.	Most mechanistic support is extrapolated from broader radiobiology and other cancers, not osteosarcoma-specific clinical proof.	Biomarker-embedded studies should compare schedules and measure immune/myeloid correlates, ctDNA, and toxicity to distinguish biologic hypothesis generation from proven clinical benefit.
SFRT for bulky or unresectable disease (see Appendix B)	Very large tumors may not be suitable for uniform ablative treatment, and SFRT may offer a pragmatic strategy for symptom relief, local regression, or bridge-to-resectability approaches.	Current support is mainly from sarcoma-general and SFRT literature, with little osteosarcoma-specific evidence.	Prospective studies should standardize peak/valley reporting, define clinical states clearly, and evaluate symptom response, wound/bone toxicity, and conversion-to-resectability.

## Appendix B

For readers seeking a concise supplementary synthesis of this pragmatic bulky disease perspective, a brief summary is provided in this appendix with a focus on SFRT.

Spatially fractionated radiation therapy (SFRT) refers to intentional intratumoral dose heterogeneity, historically associated with GRID approaches and more recently with lattice-style techniques [42,43,44,45,46,47,48,49,50,51,72]. Its relevance to this review is practical rather than doctrinal: SFRT may be most useful in bulky, symptomatic, or morbidity-prohibitive disease states where uniform dose escalation is not feasible. Although proposed mechanisms include bystander signaling, vascular disruption, and immune remodeling, these remain incompletely validated and should be regarded as hypotheses to be tested prospectively rather than assumed advantages.

- Most relevant use-case: bulky or unresectable disease in which the dominant goal is palliation, local regression, or bridge-to-resectability.
- Key reporting variables: peak dose, valley dose, peak-to-valley relationship, vertex geometry, and integration with external beam radiotherapy.
- Key cautions: heterogeneous technique, limited standardization, uncertain biologic reproducibility, and toxicity concerns including wound, skin, and bone complications.
- Research priority: prospective state-based trials with standardized dosimetry, patient-reported outcomes, local control endpoints, and feasible biomarker sampling.

A review of biologically directed radiotherapy concepts for ‘state-based radiobiotherapy’ in the context of an educational framework would be incomplete without a mention of the importance of educating future generations of clinicians and researchers on evolving radiobiology, cancer biology, and radioimmunobiology [23,70].
